# The relationship between sleep quality and occupational fatigue in endoscopy nurses: mediating role of positive coping style

**DOI:** 10.3389/fpubh.2024.1437659

**Published:** 2024-07-04

**Authors:** Zhi Zeng, Sumei Zhou, Guiqiong Xie, Yazhi He, Jing Ling

**Affiliations:** ^1^Department of Gastroenterology, Deyang People’s Hospital, Deyang, Sichuan, China; ^2^Department of Neurosurgery, Deyang People’s Hospital, Deyang, Sichuan, China

**Keywords:** endoscopy nurses, occupational fatigue, sleep quality, positive coping style, mediating effect

## Abstract

**Background:**

Nursing occupational fatigue has emerged as a critical issue affecting the safety and health of nurses. This phenomenon not only impairs nurses’ performance and mental well-being but also poses risks to patient safety and the quality of care provided. This study focuses on endoscopic nurses to explore the mediating role of positive coping styles between sleep quality and occupational fatigue, aiming to identify effective strategies to alleviate fatigue, thereby improving the work environment and enhancing healthcare quality.

**Methods:**

From July to August 2023, a cross-sectional design was used to select 258 endoscopy nurses from 25 top-three hospitals in 14 cities across 5 provinces in China. Data was collected through general information questionnaires, Fatigue assessment instrument, Pittsburgh sleep quality index, and Simple Coping Style Questionnaire. A structural equation model of sleep quality – positive coping style – occupational fatigue was constructed using Amos 26.0, and Bootstrap was employed to test the mediating effect.

**Results:**

The results showed that the mean scores of sleep quality, occupational fatigue, and positive coping style for endoscopy nurses were 8.89 ± 4.13, 17.73 ± 5.64, and 18.32 ± 10.46, respectively. Positive coping style were negatively correlated with sleep quality and occupational fatigue (*p* < 0.001). Positive coping style partially mediated the relationship between sleep quality and occupational fatigue, with a mediating effect value of 0.253, accounting for 42.10% of the total effect.

**Conclusion:**

Sleep quality can indirectly affect the level of occupational fatigue through positive coping style. Nursing managers should enhance nurses’ positive coping skills, improve nurses’ sleep quality, and reduce occupational fatigue among nurses.

## 1 Introduction

Occupational fatigue of nurses refers to a multidimensional state in which nurses face excessive demands and stressors at work or in the environment, resulting in certain disturbances in their body, cognition and normal working ability (1).

With the rapid development of endoscopic diagnosis and treatment technology, the role functions and professional capabilities of endoscopic nurses are constantly increasing. Due to the complexity and particularity of the post, the occupational fatigue detection rate of endoscopic nurses reached 57.32%, which easily led to decreased work efficiency, increased risk of errors and accidents, and emotional exhaustion, affecting the quality of endoscopic diagnosis and treatment and patient safety (2). Research has found that sleep quality is one of the independent predictors of fatigue levels among nurses (3). Nurses with sleep disorders are 2.9 times more likely to experience occupational fatigue (4).

Positive coping style refer to the characteristic of individuals actively adjusting their cognition and behavior to adopt a positive attitude and approach to adapt to a stressful environment when adapting to stressful events (5, 6). Studies indicate that choosing positive coping styles not only represents behavioral or cognitive attempts but also effectively mitigates the adverse impact of potential or actual difficulties on physical and mental health (7). A cross-sectional survey conducted in China illustrates the pivotal role of positive coping styles in regulating occupational stress and promoting occupational health, significantly influencing individual well-being and mental health (8). Specifically, nurses employ positive coping style to manage work stress, enhance psychological capital, and consequently improve their mental health (9). Research further reveals a negative correlation between positive coping and sleep quality (10), while highlighting its positive effect on reducing levels of occupational fatigue (11). Therefore, the sleep quality of endoscopic nurses is likely influenced by their active coping styles, subsequently impacting their occupational fatigue.

Currently, there is a lack of research on the interaction mechanisms among positive coping style, sleep quality, and occupational fatigue, with even fewer studies focusing on endoscopy nurses. This study hypothesizes that endoscopy nurses could reduce the occurrence of occupational fatigue by improving their sleep quality, thereby encouraging them to adopt positive coping style. If the hypothesis is confirmed, nursing managers could implement targeted interventions to guide nurses in adopting positive coping style, thereby preventing the occurrence of occupational fatigue.

## 2 Materials and methods

### 2.1 Study design and participants

**Figure 1:**
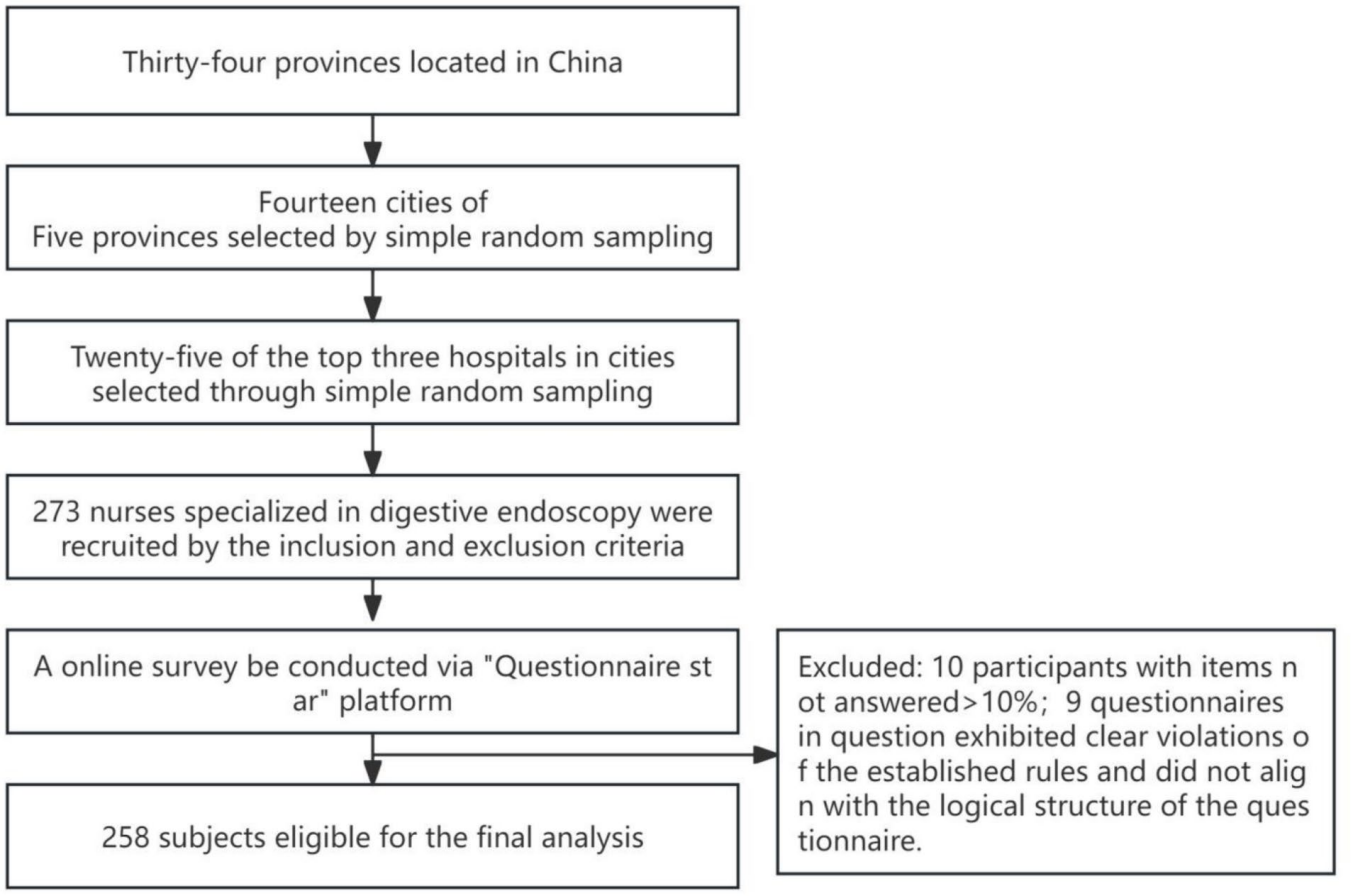
The process of participant selection.

From July to August 2023, a cross-sectional design was employed to extract 14 cities from a random sample of five provinces in China. These cities were selected to represent the 34 provinces, and 25 of the top three hospitals were randomly selected from each city. Finally, 273 endoscopic specialist nurses were randomly recruited. A total of ten participants were excluded due to incomplete responses, and at least 10 % of the items were not answered. After excluding nine questionnaires that exhibited discernible patterns or illogical responses, 258 subjects were deemed eligible for the final analysis, resulting in an effective response rate of 96.63%. Figure 1 showed the process of participant selection.

The inclusion criteria were the following: having worked in the endoscopy specialty for 1 year or more, being able to practice independently, and providing informed consent with a willingness to participate in the survey.

Exclusion criteria for this study include, Nurses who practice, study or retire; Absent nurses who are on leave or vacation; Accompanied by sleep apnea syndrome.

The sample size was estimated using the Kendall sample size estimation method (12), which recommends a sample size of 5 to 10 times the number of variables. Considering a 20% rate of invalid questionnaires, the required sample size was determined to be between 125 and 250 participants. Ultimately, this study included a total of 258 participants.

### 2.2 Ethical considerations

The study approved by the Ethics Committee of Deyang people’s Hospital (No. 2021-04-056-K01), and the personal information of participants was anonymously treated for privacy and confidentiality.

### 2.3 Research tools

#### 2.3.1 General information survey form

The questionnaire was designed based on the summary analysis of domestic and foreign literature and expert consultation. Including age, gender, education level, marital status, professional title, years of working in endoscopy specialty, daily working hours, and weekly working days.

#### 2.3.2 Fatigue assessment instrument

Fatigue Assessment Instrument (FAI) was developed by Joseph et al. (13) and Sinofication (Chinese translation and adaptation) was done by Wang et al. (14). The FAI includes 4 dimensions with a total of 29 items: global fatigue severity, situation specificity, consequences of fatigue and responsiveness to rest/sleep,. It uses a Likert 7-point scale for scoring, where each item score ranges from “completely disagree” to “completely agree”, corresponding to 1 to 7 points. The score for each dimension is calculated by averaging the scores of all items within that dimension, and the total score is the sum of the scores of the four dimensions, ranging from 4 to 28 points. Higher scores indicate more severe fatigue. The Cronbach’s α coefficient of this scale ranges from 0.70 to 0.92, and in this study, the Cronbach’s α coefficient ranges from 0.763 to 0.974.

#### 2.3.3 Pittsburgh sleep quality index

The Pittsburgh Sleep Quality Index (PSQI) was developed by Buysse et al. (15) and translated and revised by Lu et al. (16). The PSQI comprises 7 dimensions with a total of 24 items, including subjective sleep quality, sleep latency, sleep duration, sleep efficiency, sleep disturbances, use of sleeping medications and daytime dysfunction. It uses a Likert 4-point scale (0 to 3 points) for scoring. The total score ranges from 0 to 21 points, with higher scores indicating poorer sleep quality. A score greater than 7 suggests the presence of sleep disturbances. The Cronbach’s α coefficient for this scale is 0.845, and in this study, the Cronbach’s α coefficient is 0.773.

#### 2.3.4 Simple coping style questionnaire

The Simple Coping Style Questionnaire (SCSQ) was developed by Folkman et al. (17) and was translated and revised by Xie (18). The SCSQ comprises 20 items divided into two dimensions: positive coping and negative coping. It uses a Likert 4-point scale (0 to 3 points), ranging from “not taking” to “frequently taking” corresponding to 0 to 3 points, respectively. Each dimension is scored independently. In this study, only the positive coping dimension was used to assess the positive coping scores of endoscopy nurses. The total score for this dimension ranges from 0 to 36 points, with higher scores indicating a greater tendency to adopt a positive coping style. The Cronbach’s α coefficient for this scale is 0.890, and in this study, the Cronbach’s α coefficient is 0.950.

### 2.4 Data collection

The researchers contacted the head nurses of endoscopy centers (rooms) in 25 top-three hospitals for assistance in distributing questionnaires. The survey was conducted online through the WeChat group for endoscopy nursing using the “Questionnaire Star” platform. The questionnaire included standardized instructions explaining the purpose, significance, and important points of the study. Participants were informed that they could voluntarily and anonymously complete the survey, with all items required to be answered. Each IP address or WeChat ID was restricted to one submission, and the submission time was set to 10 min. After the survey period ended, the researchers exported the data. Two researchers independently inputted and checked the data, excluding any questionnaires with clearly patterned responses or illogical answers.

### 2.5 Statistical analyses

SPSS 27.0 was used for analysis, Descriptive statistical methods of frequency and percentage were used to analyze the general data of endoscopic nurses, The scores of sleep quality, occupational fatigue and active coping style were analyzed by means of mean and standard deviation after normal distribution test. Pearson correlations were used to analyze correlations between factors. The Harman single factor test was used to Common Method Bias Test. Amos 26.0 was used to establish the structural equation model, and Bootstrapping was utilized to test for mediating effects. The test level was α = 0.05.

## 3 Results

### 3.1 General data of endoscopic nurses and univariate analysis of occupational fatigue in endoscopic nurses with different characteristics

A total of 258 endoscopic nurses were included in this study. The comparison of occupational fatigue scores among nurses with different genders, professional titles, years of service, daily working hours, and weekly working days showed statistically significant differences (Table 1).

**Table 1:** General data of endoscopic nurses and univariate analysis of occupational fatigue in endoscopic nurses with different characteristics (*n* = 258).

Variables	*n* (%)	*x̅* ± s	t/F	*p*
Gender				
Male	56 (21.71)	16.31 ± 5.72	4.556	0.034
Female	202 (78.29)	18.12 ± 5.56		
Age				
20–30	74 (28.68)	18.08 ± 5.89	0.150	0.930
30–40	151 (58.53)	17.55 ± 5.50		
40–50	28 (10.85)	17.77 ± 5.98		
≥50	5 (1.94)	17.51 ± 5.32		
Marital status				
Not married	61 (23.64)	17.52 ± 6.00	0.514	0.599
Married	191 (74.03)	17.72 ± 5.57		
Divorced	6 (2.33)	19.97 ± 3.50		
Education level				
Diploma	59 (22.87)	16.41 ± 5.65	2.428	0.090
Bachelor’s	188 (72.87)	18.03 ± 5.65		
Master’s	11 (4.26)	19.46 ± 4.39		
Professional title				
Registered nurse	18 (6.98)	16.12 ± 5.17	2.960	0.033
Senior Nurse	130 (50.39)	18.74 ± 5.39		
Supervisor nurse	95 (36.82)	16.83 ± 5.94b		
Co-chief /chief nurse	15 (5.81)	16.56 ± 5.13		
Registered nurse				
<3 years	88 (34.11)	20.95 ± 3.41	36.482	<0.001
3–5 years	74 (28.68)	18.28 ± 5.58a		
5–10 years	63 (24.42)	15.85 ± 4.99ab		
≥10 years	33 (12.79)	11.45 ± 5.21abc		
Daily working hours				
8 h	4 (1.55)	6.73 ± 1.30	23.939	<0.001
8–9 h	208 (80.62)	16.83 ± 5.34a		
9–10 h	31 (12.02)	22.48 ± 2.71ab		
≥10 h	15 (5.81)	23.72 ± 4.13ab		
Weekly working days				
5d	139 (53.88)	16.73 ± 5.85	5.153	0.002
5.5–6d	90 (34.88)	18.41 ± 4.95a		
6–6.5d	24 (9.30)	19.57 ± 5.77a		
≥6.5d	5 (1.94)	24.16 ± 2.34ab		

a: Compared with layer 1, *p* < 0.05; b: Compared with layer 2, *p* < 0.05; c: Compared with layer 3, *p* < 0.05.

### 3.2 The scores of occupational fatigue, sleep quality, and positive coping styles in endoscopy nurses

The total scores for occupational fatigue, sleep quality, and positive coping styles in endoscopy nurses were (17.73 ± 5.64) points, (8.89 ± 4.13) points, and (18.32 ± 10.46) points, respectively. The detailed scores for each dimension and item can be found in Table 2.

**Table 2:** The scores of occupational fatigue, sleep quality, and positive coping styles in endoscopy nurses (*n* = 258).

Variables	Dimensions/items	Score range	Score situation
Total score of sleep quality	7	0–21	8.89 ± 4.13
Subjective sleep quality	2	0–3	2.33 ± 0.23
Sleep latency	1	0–3	0.99 ± 0.83
Sleep duration	9	0–3	1.09 ± 0.57
Sleep efficiency	3	0–3	1.04 ± 0.64
Sleep disturbances	1	0–3	0.60 ± 0.95
use of sleeping medications	2	0–3	1.45 ± 0.01
Daytime dysfunction	1	0–3	1.39 ± 0.95
Total occupational fatigue score	4	4–28	17.73 ± 5.64
Global fatigue severity	11	1–7	4.41 ± 1.56
Situation specificity	6	1–7	4.30 ± 1.53
Consequences of fatigue	3	1–7	4.46 ± 1.49
Responsiveness to rest/sleep	2	1–7	4.56 ± 1.56
Total positive coping style score	12	0–36	18.32 ± 10.46

### 3.3 Correlation analysis of occupational fatigue, sleep quality, and positive coping style in endoscopic nurses

Pearson correlation analyses for the main variables are presented in Table 3. The results showed that occupational fatigue was positively correlated with sleep quality (*r* = 0.521, *p* < 0.001), negatively correlated with active coping style (*r* = −0.572, *p* < 0.001), and negatively correlated with sleep quality and positive coping style (*r* = −0.505, *p* < 0.001).

**Table 3:** Correlation analysis of occupational fatigue, sleep quality and positive coping style in endoscopic nurses (*n* = 258).

Variables	Positive coping style	Occupational fatigue	Positive coping style
Sleep quality	1		
Occupational fatigue	0.521**	1	
Positive coping style	−0.505**	−0.572**	1

** At a 0.01 level (double tails), the correlation was significant.

### 3.4 Common method bias test

The Harman single factor test was used to perform the common method bias test for all variables. The results show that there are 10 factors with feature root >1, and the variance explained by the first factor is 38.290%, which is less than the critical value of 40%, indicating that there is no serious common method bias in this study.

### 3.5 Analysis of the mediating effect of positive coping style on the relationship between sleep quality and occupational fatigue in endoscopy nurses

**Figure 2:**
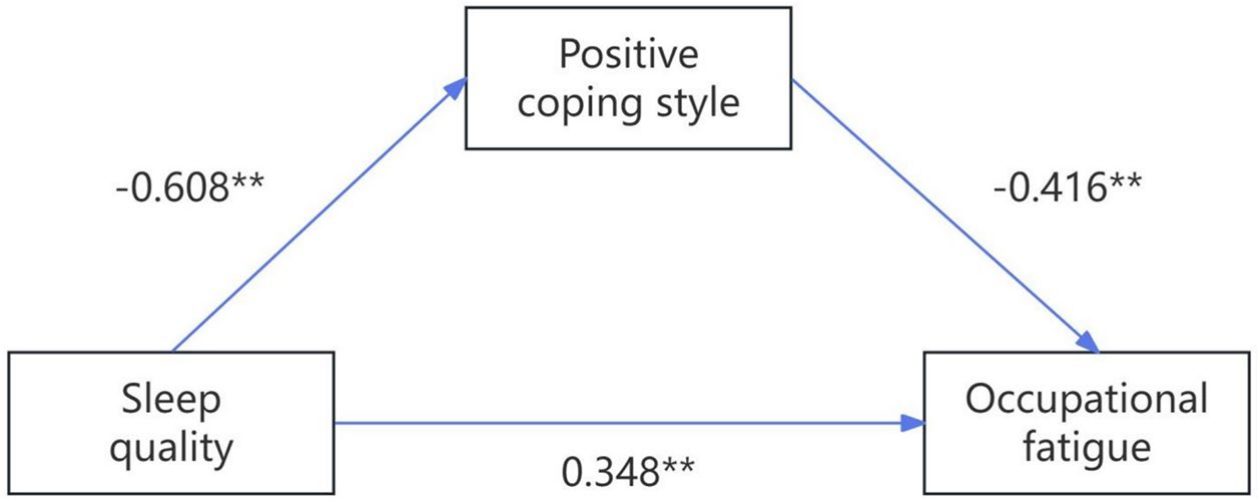
Mediating model of occupational fatigue, sleep quality and active coping style in endoscopic nurses. ** Indicates that *p* < 0.01 and *** states that *p* < 0.001.

Amos26.0 software was used to build a structural equation model with positive coping style as the mediating variable, sleep quality as the independent variable, and occupational fatigue as the dependent variable. Maximum likelihood method was used to fit the model and modify the model, and finally the model was built, as shown in Figure 2. The model fitting results such as Table 4. All parameters are within the ideal range, indicating good model fit.

**Table 4:** Structural equation model fitting index (standardized).

Project	Statistical tests	Suggested reference standards	Test results	Fit or not
Parsimony fit index	CMIN/DF	1–5	1.910	YES
Absolute fit indices	RMSEA	<0.05 fit was good, <0.08 fit was acceptable	0.060	YES
	GFI	>0.90	0.932	YES
	AGFI	>0.90	0.903	YES
Relative fit indices	NFI	>0.90	0.932	YES
	IFI	>0.90	0.932	YES
	CFI	>0.90	0.932	YES

Bootstrap method was used to conduct 5,000 repeated samples with a confidence interval of 95% (excluding 0) to test the mediating effect of active coping styles on sleep quality and occupational fatigue. The results showed as follows: Sleep quality negatively predicted positive coping style (β = −0.608, *p* < 0.001), positive coping style negatively predicted occupational fatigue (β = −0.416, *p* < 0.001), and sleep quality positively predicted occupational fatigue (β = 0.348, *p* < 0.001). The positive coping style played a partial mediating role in the relationship between sleep quality and occupational fatigue. The mediating effect value was 0.253, and the total effect value was 0.601, accounting for 42.10% of the total effect, as shown in Table 5.

**Table 5:** Bootstrap analysis of the mediating effect of positive coping style between sleep quality and occupational fatigue.

Project	Standardized effect value	SE	95%CI	*p*	Relative effect value (%)
Indirect effect	0.253	0.045	0.172 ~ 0.353	0.000	42.10%
Direct effect	0.348	0.082	0.183 ~ 0.510	0.000	57.90%
Total effect	0.601	0.055	0.485 ~ 0.699	0.000	100.00%

## 4 Discussion

### 4.1 Analysis of the current status of sleep quality, positive coping style, and occupational fatigue in endoscopy nurses

The results showed that the sleep quality score of endoscopic nurses was (8.89 ± 4.13), slightly higher than that of emergency department (ED) nurses (8.2 ± 3.9) and general nurses (19, 20), indicating that the sleep quality of endoscopy nurses is not optimistic. The specialty’s work involves strong technical expertise, high precision in surgical assistance, large workloads, and overburdened work hours. These factors negatively impact their sleep. Compared to general nurses, frequent close contact with patients increases the likelihood of workplace violence compared to general ward nurses (21), which can easily lead to lower job satisfaction in career development, generating anxiety and stress, and consequently reducing sleep quality. In contrast, ED nurses face more night shifts and irregular work schedules but benefit from a team-based work model that provides significant peer support, alleviating work-related stress and improving sleep quality. Managers could mitigate the sleep conditions of endoscopy nurses by implementing positive measures such as strengthening specialized knowledge training, introducing flexible scheduling, providing team support, and reasonably allocating human resources.

The positive coping style score of endoscopic nurses was (18.32 ± 10.46), which was at the medium level, lower than Wu et al. (22). This may be related to the high proportion of young and middle-aged married women and junior titleholders included in this study. The relatively younger age structure and lack of work experience mean fewer coping measures and limited forms. When bearing the responsibility of caring for family members and handling complex surgical procedures, they may need to invest more energy and time to adapt, making it difficult to balance and switch between work and life, thus lacking the ability to handle problems positively. In terms of age structure and work experience, endoscopic nurses are younger and have less work experience compared to subjects in other studies, which is associated with lower positivity in coping styles (23). The influence of marital status and family responsibilities on coping styles should not be ignored. Married endoscopic nurses also face balance pressure. Moreover, professional titles significantly impact coping strategies. Lower professional titles may imply reduced recognition and career growth opportunities, leading to decreased job satisfaction and motivation, and consequently, less effective coping mechanisms (24). Managers should pay close attention to the cultivation of positive coping style among endoscopy nurses, such as improving the work environment, providing mental health support, offering career development opportunities, and enhancing team building to promote the adoption of a positive attitude in coping with stressful events.

The occupational fatigue score was (17.73 ± 5.64), and the detection rate of occupational fatigue was 60.9%, which was at the medium level, higher than the results of Fatma et al. (25). This may be related to the heavy workload in endoscopy, lack of adequate rest and recovery time, the mismatch of endoscopy nurses’ role functions and practical abilities, low levels of professional respect, relatively weak sense of professional value, and long-term exposure to environments with patients’ blood, body fluids, chemical disinfectants, and radiation (26, 27). Comparing workloads, endoscopy nurses often face intense and demanding tasks similar to those in emergency departments and intensive care units, but with distinct procedural challenges (28, 29). Rest and recovery time is often insufficient, paralleling findings in other high-stress nursing environments (30). Professional respect and value are crucial; studies in various nursing fields indicate that higher professional respect correlates with lower fatigue levels (31). This suggests that managers should implement targeted coping strategies to alleviate work stress, enhance training for professional identity, improve specialized practical capabilities, and reduce occupational fatigue in endoscopy nurses.

### 4.2 The correlation between sleep quality, active coping style, and occupational fatigue in endoscopic nurses

This study showed that the sleep quality and scores of all dimensions of endoscopic nurses were positively correlated with occupational fatigue, among which Subjective sleep quality (*r* = 0.428, *p* < 0.001) and sleep efficiency (*r* = 0.411, *p* < 0.001) were the most correlated with occupational fatigue, indicating that the poorer the sleep quality of the nurses, the higher their occupational fatigue scores, which is consistent with the findings in literature (32). Long working hours, high stress, and irregular work schedules can disrupt the individual’s biological clock and normal sleep–wake cycle, leading to difficulties falling asleep and decreased sleep quality (33). Prolonged insufficient sleep quality can result in sluggish thinking, distraction, diminished cognitive function, memory decline, and increased feelings of fatigue and tension, which can trigger and exacerbate occupational fatigue. According to the effort-recovery (E-R) model (34), when an individual’s psychophysiological system is activated by work demands and accumulated fatigue, recovery becomes a relaxing process, enabling the individual to return to baseline from a fatigued state. Evidence from empirical studies indicates that reducing working hours, avoiding overtime, increasing the number of nursing positions, extending night shift rotation cycles, and maintaining regular sleep schedules can effectively improve nurses’ sleep quality and reduce recovery time from fatigue (35, 36). High-quality sleep not only helps restore physical and mental energy, enhance work efficiency and creativity, but also maintains emotional stability and work-life balance, thereby reducing the occurrence and severity of occupational fatigue. These measures have a significant positive impact on the overall health and job performance of nurses. Managers can promote fatigue recovery by reasonably arranging work, avoiding overwork and interruptions during non-working hours, and providing ample rest and sleep time after work.

Positive coping style was negatively correlated with occupational fatigue. Existing research indicates that when nurses experience work-related stress, maintaining a positive and optimistic attitude, adopting healthy lifestyle habits, and actively seeking family and social support are effective to alleviate fatigue and promote physical and mental health (37, 38). According to the stress and coping model (7), after experiencing external negative stimuli, individuals can actively seek social support and assistance to reduce the impact of negative events and positively adjust to cope with stress. Positive coping style encourage endoscopy nurses to take proactive measures to solve problems and effectively manage themselves when facing stressors that lead to occupational fatigue. By cultivating healthy lifestyles and improving sleep quality, these strategies help reduce occupational fatigue (39, 40). It suggests that managers should strengthen the training of nurses in positive coping skills to mitigate the negative impacts of occupational fatigue and poor sleep quality.

### 4.3 The mediating effect of positive coping style on sleep quality and occupational fatigue in endoscopy nurses

The results of mediation analysis showed that active coping strategies played a partial mediating role between sleep quality and occupational fatigue. It is suggested that sleep quality can directly influence occupational fatigue, as well as indirectly influence it through positive coping style. According to the cognitive appraisal theory of stress (41, 42), high work pressure and workload can induce anxiety, depression, and other negative psychological symptoms. Effective coping strategies influence how individuals perceive and respond to these stressors, thereby affecting their adaptability and health outcomes.

When suffering from stressors affecting sleep disorders, endoscopic nurses adopt positive coping methods, transform work pressure into internal motivation, and actively self-adjust their own behaviors and emotions to improve sleep quality, thereby indirectly reducing occupational fatigue (43).

These findings imply that managers should prioritize understanding the psychological states of nurses and promoting the adoption of positive coping styles. Strategies such as specialized lectures, group interventions, and stress management training can empower nurses to effectively appraise and manage stressors, ultimately reducing occupational fatigue and promoting overall well-being.

## 5 Study limitations

In this study, we evaluated the relationship and interaction between positive coping style, sleep quality, and occupational fatigue. We found that positive coping style partially mediated the relationship between sleep quality and occupational fatigue among endoscopy nurses. However, this study has several limitations. First, limited by human resources, material resources, specialty bases and other objective conditions, this study only investigated endoscopic nurses in some provinces and cities in China, the research results may be biased by regional and economic development level and other factors. Second, this study only used the positive coping style from the Simple Coping Style Questionnaire as a mediating variable. The sleep quality and occupational fatigue of digestive endoscopy nurses may also be influenced by negative coping or other variables. In future research, we plan to expand the study population and geographical scope, incorporate cross-cultural comparisons and objective measurement tools, and further control for potential confounding factors. Additionally, we intend to conduct longitudinal studies to further validate the results.

## Data availability statement

The original contributions presented in the study are included in the article/supplementary material, further inquiries can be directed to the corresponding author.

## Ethics statement

The studies involving humans were approved by the Ethics Committee of Deyang people’s Hospital. The studies were conducted in accordance with the local legislation and institutional requirements. The participants provided their written informed consent to participate in this study.

## Author contributions

ZZ: Data curation, Project administration, Writing – original draft, Writing – review & editing. SZ: Methodology, Visualization, Writing – original draft, Writing – review & editing. GX: Data curation, Investigation, Methodology, Supervision, Validation, Visualization, Writing – review & editing. YH: Funding acquisition, Supervision, Writing – review & editing. JL: Formal analysis, Methodology, Writing – review & editing.
